# Effect of gut microbiota modulation on feeding tolerance of enterally fed critically ill adult patients: a systematic review

**DOI:** 10.1186/s13643-021-01633-5

**Published:** 2021-04-02

**Authors:** Najmeh Seifi, Ali Jafarzadeh Esfahani, Alireza Sedaghat, Reza Rezvani, Majid Khadem-Rezaiyan, Mohsen Nematy, Mohammad Safarian

**Affiliations:** 1 Department of Nutrition, Medical School, Mashhad University of Medical Sciences, Mashhad, Iran; 2 Department of Anesthesiology, Mashhad University of Medical Sciences, Mashhad, Iran; 3 Department of Community Medicine, Medical School, Mashhad University of Medical Sciences, Mashhad, Iran; 4 Metabolic Syndrome Research Center, Mashhad University of Medical Sciences, Mashhad, Iran

**Keywords:** Prebiotics, Probiotics, Synbiotics, Gut microbiota, Feeding tolerance, Critical care

## Abstract

**Purpose:**

The objective of this systematic review was to evaluate the effect of pre-, pro-, and synbiotics on feeding tolerance of enterally fed critically ill adult patients.

**Methods:**

MEDLINE, Science Direct, Web of Knowledge, and the Cochrane Central Register of Controlled Trials were searched up to November 2019. English language randomized controlled trials reporting the effect of pre, pro or synbiotics on the feeding tolerance of enterally fed critically ill adult patients were included.

**Results:**

Overall, 15 papers were selected for review. Among six studies reporting the energy intake, only two studies showed significantly higher energy intake in the prebiotic-receiving groups. Among four RCTs reporting frequency or time to achieve the target calorie, only one found a significant effect of probiotics to reduce the time to achieve a target dose of calorie. About the prevalence or duration of diarrhea, 7 out of 12 RCTs reported a beneficial effect. All but one study found no beneficial effects for gut microbiota manipulation on clinical endpoints including length of stay (LOS) in hospital and intensive care unit (ICU).

**Conclusion:**

It should be noticed that the heterogeneity in study designs, product format, and ICU patient populations makes it difficult to draw any general conclusion. Overall, it seems that pre, pro, or synbiotics have no significant beneficial effect on feeding tolerance and clinical endpoints in critically ill adults, but they may reduce the prevalence or duration of diarrhea.

**Supplementary Information:**

The online version contains supplementary material available at 10.1186/s13643-021-01633-5.

## Background

Critical illness can cause hypermetabolism and hypercatabolic state that quickly depletes nutritional reserves, alters immune function, and predisposes individuals to morbidities and mortality [1, 2]. Critically ill patients are also likely to experience severe changes in gut function. These changes are due to alterations in gut muscle contractions, secretion, and absorption. Gut microbiota disturbances and epithelial barrier disintegration are also involved [3–5]. In this situation, early-onset and the proper amount of nutrition support are of great importance [6]. Enteral nutrition (EN) is regarded as the favored root of nutrition support, because it protects the gut barrier, modulates immune responses, and leads to a faster return of gut function. However, many critical care patients cannot receive EN due to tolerance problems [4, 6].

Enteral feeding intolerance is a common problem among critical care patients. It is often defined as either or both of the following conditions: reduced delivery of EN and presence of gastrointestinal (GI) symptoms, including diarrhea, vomiting, regurgitation, abdominal distention, and high gastric residual volume (GRV) [7]. Feeding intolerance often results in failure to achieve the target nutritional dose as well as increased risk of pneumonia and intensive care unit (ICU) stay [8]. Factors associated with feeding intolerance in critically ill patients include stress-induced hyperglycemia; hormonal disturbances (including high levels of cholecystokinin (CCK), and peptide YY (PYY), and low levels of motilin); administration of sedatives, analgesics, and vasopressor agents; and disturbances in gut microbiota. These factors finally result in gastrointestinal dysfunction and manifest as feeding intolerance [9].

Gut microbiota manipulation can affect enteral feeding tolerance and energy homeostasis through several mechanisms. Administration of pre, pro, or synbiotics are different ways of gut microbiota manipulation. Probiotics are live microorganisms that have beneficial health effects if administered in optimum amounts. Prebiotics are non-digestible oligosaccharides that promote growth and/or activity of specific bacteria in the gut. Synbiotics are products with a combination of probiotics and prebiotics [10]. Altering gut muscle contractions, secretion, and absorption [11–13]; regulating glucose homeostasis [14, 15]; and affecting hormonal and immune responses, host metabolism, and feeding behavior [16] are known mechanisms by which gut microbiota modulation can affect feeding tolerance and energy homeostasis.

Recently, the relationship between gut microbiota and nutrition, especially in critically ill patients has been attracting considerable interest. Many studies have reported the effect of pre, pro, or synbiotics on EN volume, energy intake, or EN-associated complication. Nevertheless, to the best of our knowledge, no systematic review or meta-analysis has been conducted to evaluate the effect of pre-, pro-, and synbiotics on feeding tolerance of enterally fed critically ill adult patients.

## Methods

This systematic review was consistent with the Preferred Reporting Items for Systematic Reviews and Meta-Analysis (PRISMA) statement (Additional file 1) [17].

### Search strategy

A systematic search of randomized controlled trials published until November 10, 2019, was independently conducted by two authors (NS, AJE) on MEDLINE (via PubMed), Science Direct (via Scopus and Embase), Web of Knowledge (via Web of Science), and the Cochrane Central Register of Controlled Trials (via Cochrane Library). The search strategy was designed in accordance with the database orientations using Boolean operators (AND, OR), parenthesis, quotation marks, and asterisks. The following search strategy was used in MEDLINE: (“critical*” OR “critical care” OR “critical illness” OR “critically ill” OR “critically unwell” OR “severely unwell” OR “severely ill” OR “intensive care” OR “ICU” OR “CCU") AND (“tube feeding” OR “enteral*” OR “enteral feeding” OR “enteral nutrition” OR “force-feeding” OR “nasogastric*” OR “nasoduodenal*” OR “nasojejunal*”) AND (prebiotic* OR probiotic* OR synbiotic* OR symbiotic) NOT (child OR pediatric OR infant OR preterm OR neonate) OR ((“Enteral Nutrition”[Mesh]) AND (“Critical Care”[Mesh] OR (“tolerance” OR “intolerance” OR “tolerant” OR “intolerant”) OR (“diarhea” OR “diarhoea” OR “distension” OR “distent*”)) AND (“Probiotics”[Mesh] OR “Synbiotics”[Mesh] OR “Prebiotics”[Mesh])). Language restriction was applied to select articles in English. Furthermore, a manual reference check was conducted on the identified articles to find further relevant studies.

### Screening and eligibility of records

The Population, Intervention, Comparison, Outcome, and Study design (PICOS) strategy was used to identify inclusion criteria. The inclusion and exclusion criteria are presented in Table 1. In summary, RCTs that were published in English language; included adult critically ill patients undergoing tube feeding; administered pre, pro, or symbiotics in the intervention group and placebo or routine care to the control group; and assessed enteral feed volume, time to reach full enteral nutrition, the prevalence of feed intolerance, and related GI complications were included in the review. Studies that included patients who received partial EN or in vitro studies were excluded. Based on the review protocol, author of studies that seemed to include other outcomes in the study but failed to report the results were contacted and asked for the missing data to avoid reporting bias. Unfortunately, the authors either did not respond to the email in the designated time of the review or the data were not available for further analysis.

**Table 1:** PICOS criteria for inclusion and exclusion of criteria

Parameter	Inclusion criteria	Exclusion criteria
Population	Adult tube-fed critically ill patients	Partial EN
Intervention	Supplementation with pre, pro, or synbiotics	
Comparison	Placebo or nothing	
Outcome	Enteral feed volume, Time to reach full enteral nutrition, the prevalence of feed intolerance and related GI complications (diarrhea, distention, high residual volume)	
Study design	Randomized controlled trials	In vitro studies

*EN* Enteral nutrition; *GI* Gastrointestinal

The title and abstract of all identified articles were independently screened by two authors (NS, AJE). Randomized controlled trials that assessed the effect of pre, pro, and synbiotics on feeding tolerance in tube-fed critically ill patients were selected. The full text of selected articles was read and assessed regarding compliance with established eligibility criteria. Discrepancies were resolved by discussion with a third researcher (RR).

### Data extraction and synthesis

The following variables were considered in data extraction: title, authors, year, country, study aim, population features (sex, age, number of participants), experimental design, intervention (the composition of prebiotic, probiotic and synbiotic, dose, and timing of administration), and main results. The *I*^*2*^ statistics was used to assess heterogeneity of the studies. The analysis indicated that the heterogeneity of the studies was high (74%). The source of heterogeneity in the included articles in this systematic were study population, inclusion and exclusion criteria, intervention, duration, and reported outcomes. Due to the heterogeneity, further statistical comparison was not possible. Therefore, vote counting was the method used for data synthesis. The vote counting strategy was performed to sum up the findings of the included studies [18]. Vote counting was performed based on the risk of bias, sample size, and the statistical significance of the findings of the included studies. The harvest plot was then used to present the findings of the vote counting with colors representing studies, bar width representing sample size, and bar height representing study quality (JADAD score).

### Risk of bias assessment

The 5-point JADAD score was used independently by two authors (NS, AJE) to assess the quality of included studies. Discrepancies were resolved by discussion with a third researcher (MKR). The five domains of the JADAD score included being randomized, appropriately describing of randomization, being double-blind, appropriately describing blinding, and explanation of withdrawal and dropouts.

### Clinical outcomes

In this study, feeding intolerance was defined as either or both of the following conditions: reduced delivery of EN and presence of GI symptoms, including diarrhea, vomiting, regurgitation, abdominal distention, and high GRV. So, the clinical outcomes of interest were enteral feed volume; energy intake, which correlates with feed volume; time to reach full enteral nutrition; the prevalence of feed intolerance; and related GI complications (diarrhea, distention, high residual volume). Diarrhea was considered as a sole outcome and was assessed based on the duration of diarrhea or frequency of defecation in patients. Length of stay (LOS) in the ICU or hospital was investigated as a secondary outcome. The treatment effect was assessed based on the presence of a significant difference in the measurement of the outcome variables between the intervention and control groups.

## Results

### Study identification and selection

**Fig. 1:**
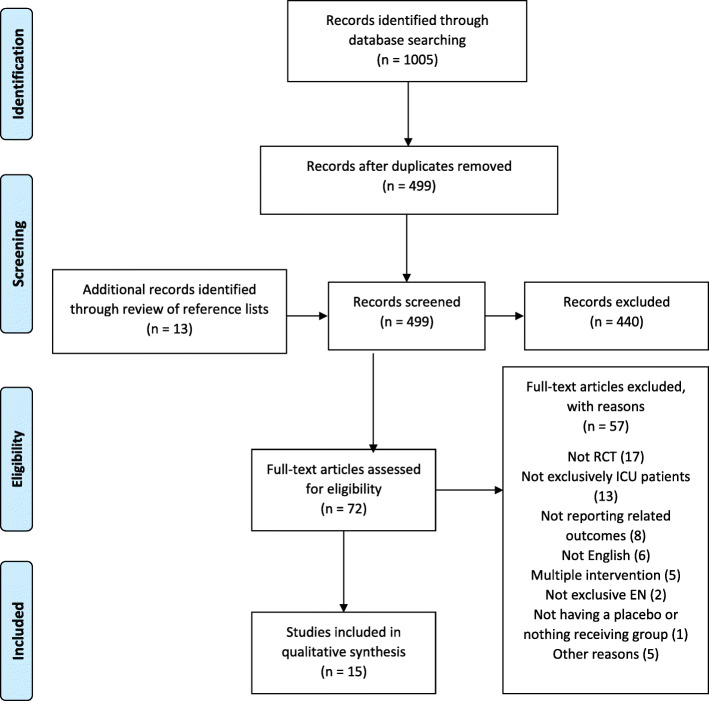
Flow diagram of the literature search process

**Fig. 2:**
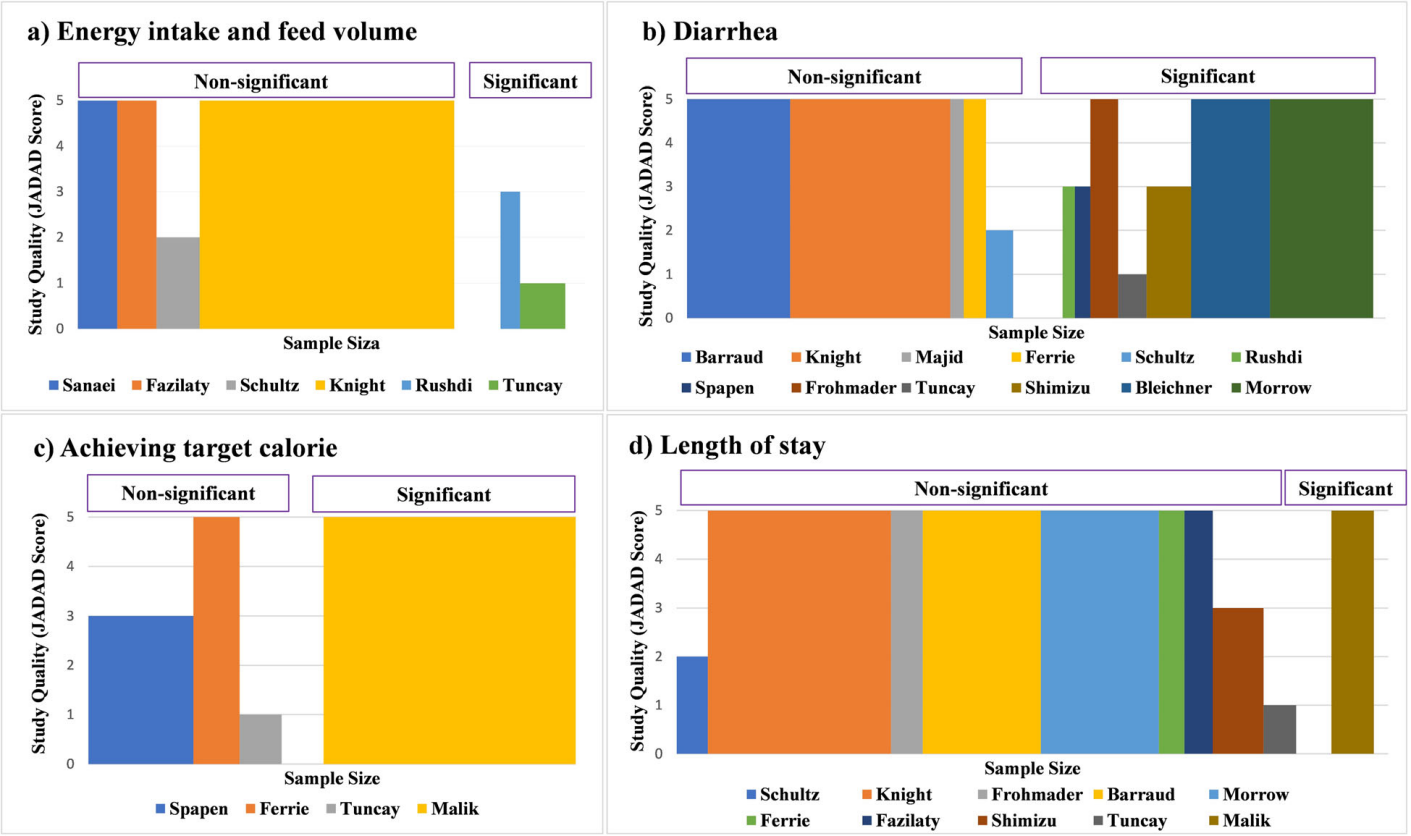
Harvest plot for the difference between intervention and control groups in terms of achieving target calorie (**a**), energy intake and feed volume (**b**), length of stay (**c**), and diarrhea (**d**)

A total of 93 relevant articles were identified through a database search and a review of reference lists of related articles. We excluded 78 articles due to the following reasons: 21 were conference abstracts, or the full text was not available, 17 were not RCTs, 13 did not exclusively enroll ICU patients, 6 were not in English, 8 did not report relevant outcomes, 5 had multiple interventions, 2 did not include exclusive EN, 1 did not have a placebo receiving control group, and 5 due to other reasons, e.g., irrelevant intervention or inconsistency in results. Finally, 15 RCTs, with a total of 1139 patients, were included (Fig. 1). As the articles included in this systematic review were heterogeneous in population, inclusion and exclusion criteria, intervention, duration, and reported outcomes, further statistical comparison was not possible. The mean JADAD score of all trials was 4.2. The minimum JADED score was 1 point for randomization. A summary of the findings of the studies is presented in Table 2. The harvest plot was presented for each outcome assessed with the orange color representing non-significant finding of the study and blue color representing significant finding. The length of the bars represents JADAD score for each study (Fig. 2).

**Table 2:** Randomized controlled trials evaluating the effect of pre, pro or synbiotics on feeding tolerance of enterally fed critically ill patients

Author, year	Population	Design	JADAD score	EN protocol	Type of intervention		
					Delivery vehicle	Intervention /dose/duration	control
Bleichner et al., 1997 [17]	ICU patients *n*=128	Parallel	5	NR	NGT or jejunostomy	*EN (intact protein standard diet without fiber or lactose) + Saccharomyces boulardii /*500 mg four times a day/limited to 21 days or to the withdrawal of EN	*EN (intact protein standard diet without fiber or lactose) +* placebo
Schultz et al., 2000 [18]	ICU patients *n*=44	Parallel	2	NR	Tube feeding	Fiber containing formula+ pectin or fiber-free formula +pectin/ 20ml, twice daily/ 6 days	Fiber containing formula+ placebo Or fiber-free formula +placebo
Spapen et al., 2001 [19]	ICU patients with severe sepsis or septic shock *n*= 25	Parallel	3	Start: first 24h, 25cc/h. Increase 25–35 cc/h to 80% target	NGT	EN+ partially hydrolyzed guar/ 22g/l / a maximum of 21 days or to the withdrawal of EN	Fiber-free EN
Rushdi et al., 2004 [20]	ICU patients with persistent diarrhea *n*=20	Parallel	3	Start: first 18–24h. Target: 25–35 kcal/kg. First day: 50%, second day: 75%, third day: 100%	NJT	EN+ 2% soluble guar gum (Benefiber)/ 4 days	Fiber-free EN
Knight et al., 2009 [21]	ICU patients *n*= 259	Parallel	5	Start: 30cc/h; max: 80cc/h; increase or decrease according to GRV	NGT/ OGT	EN (Nutrison Energy) + Synbiotic 2000 FORTE / twice a day/ to the earliest of the following time point:28 days after admission, death or discharge	EN (Nutrison Energy) + placebo
Frohmader et al., 2010 [22]	ICU patients *n*= 45	Parallel	5	Start: first 24h, 20cc/h; increase: 20cc/4h to target. Target: 25–35 kcal/kg	NGT/ OGT/ nasojejunostomy	Fiber-free EN+ probiotic (VSL#3) /twice a day/ mean of 11.9 days	Fiber-free EN+ placebo
Barraud et al., 2010 [23]	ICU patients with MV *n*=167	Parallel	5	Starting in the first 24h, 10 kcal/kg, increase to 30–35 kcal/kg	NGT	EN + multi-strain probiotic (Ergyphilus)/ once a day/ until successful weaning (maximum of 28 days)	EN + placebo
Morrow et al., 2010 [24]	ICU patients with MV *n*=167	Parallel	5	NR	NGT	EN + probiotic (*Lactobacillus rhamnosus* GG) / twice a day/	EN+ inulin-based placebo
Ferrie and Daley, 2011 [25]	ICU patients with diarrhea *n*= 36	Parallel	5	NR	Gastric tube	Fiber containing EN+ *probiotic (*inulin-based *Lactobacillus* GG)/twice a day/ 7 days	Fiber containing EN+ placebo (inulin)
Sanaie et al., 2014 [26]	ICU patient *n*= 40	Parallel	5	Start in first 24h, 25cc/h; increase 25cc/4h to target. Target: 25–30 kcal/kg	NGT	Fiber containing EN+ probiotic (VSL#3)/ twice daily/ 7 days	Fiber containing EN+ placebo
Majid et al., 2014 [27]	ICU patients *n*= 22	Parallel	5	Energy estimation based on Schofield equation	NGT	Fiber containing EN+ additional oligofructose/inulin/ 7g per day/ 7days	Fiber containing EN+ placebo
Malik et al., 2016 [28]	ICU patients *n*= 60	Parallel	5	25 kcal/kg. start in first 24–48h, with GRV management	NGT	EN+ multi-strain probiotic/ twice a day/ 7 days	EN+ placebo
Fazilaty et al., 2018 [29]	Multiple trauma ICU patients *n*= 40	Parallel	5	Goal: 25–30 kcal/kg, 75% in the 48h	NGT	EN+ prebiotic (oat β-glucan)/ 3g per day/ 21 days	EN+ placebo (maltodextrin)
Shimizu et al., 2018 [30]	Septic ICU patients with MV *n*= 72	Parallel	3	Start: 20cc/h; increase: 20cc/h/day to target. Target: 25–30 kcal/kg	NGT	EN +multi-strain probiotic (Yakult BL Seichoyaku) 3 g per day+ prebiotic (galactooligosaccharides) 10g per day/ until EN stop	EN
Tuncay et al., 2018 [31]	Neurocritical care patients *n*=46	Parallel	1	Start: 10cc/h; increase: 10cc/8h till 20cc/h; requirement: Schofield equation+stress factor+activity factor+ ventilator support+fever+TEF	Nasofeeding, gastrostomy/PEG	EN with prebiotic content/ 21 days	EN

*NR* Not reported; *NGT* Nasogastric tube; *EN* Enteral nutrition; *ICU* Intensive care unit; *OGT* Orogastric tube; *GRV* Gastric residual volume; *VSL#3*, a single daily high dose probiotic preparation

### Effect on energy intake and feed volume

Six trials examined the effect of pre, pro, or synbiotics administration on energy intake or feed volume in critically ill patients.

#### Prebiotics

In one trial in 2000, 44 critically ill patients receiving EN and antibiotics were randomized to receive fiber-containing or fiber-free formula and pectin or placebo for 6 days. Mean energy intake ranged from 1200 kcal on day 1 to 1563 kcal on day 5. Mean energy or protein intake was not significantly different in the four study groups [19]. Rushdi et al. also evaluated the effect of guar gum enriched formula in 20 critically ill tube-fed patients with persistent diarrhea for 4 days. They showed that patients in the intervention group tolerated significantly higher formula volumes on days 1, 2, and 4. On the fourth day, the feed volume was 1775±450 ml in the intervention group compared to 1070±604 ml in the controls (*p*<0.01) [20]. In 2018, Fazilaty et al. evaluated the effect of EN containing β-glucan on inflammatory markers and clinical outcomes. They reported no significant difference in the mean tolerated calories between study groups (1710.5±117.03 kcal vs. 1718.2±182.4 kcal, *p*=0.6) [21]. Tuncay et al. compared the effect of an enteral formula enriched with prebiotic versus standard EN on nutritional parameters among 46 neurocritical ill patients. Results showed that feed volume and mean energy intake significantly increased from baseline to day 21 in both groups. Patients in the intervention group tolerated a significantly higher amount of energy and feed volume on day 1 and 21 [22].

#### Probiotics

In a trial conducted in 2014, 40 critically ill patients were randomly assigned to receive a multi-strain probiotic or placebo for 7 days. Results showed no significant difference between groups in terms of the mean energy intake (1503.75±231.6 kcal vs. 1617.5±185.51 kcal, *p*= 0.09). The percentage of patients who met energy requirements in the synbiotic and placebo groups was 84.98±3.6 and 87.24±3.92, respectively (*p*=0.06) [23].

#### Synbiotics

Knight et al. investigated the effect of enteral synbiotic on ventilator-associated pneumonia in critically ill patients. They reported an increase in the daily tolerated feed volume from days 1 to 7 in both groups. The feed volume ranged from 488.9± 622.8 ml on day 1 to 1055.6±722.6 ml on day 7 in the synbiotic group and from 360±431.7 ml to 1243.9±810.3 ml in the placebo group. There was no significant difference between the two groups regarding the mean tolerated enteral feed volume [24].

Based on the findings of the studies and the harvest plot, it can be concluded that majority of the studies indicated a non-significant effect for pre, pro, or synbiotics administration on energy intake and feed volume.

### Effect on target calorie achievement

Four trials assessed the effect of prebiotics or probiotics on the prevalence of target calorie achievement or time to receive the target calorie.

#### Prebiotics

In a trial conducted in 2001, severe sepsis or septic shock patients were randomly assigned to receive EN supplemented with partially hydrolyzed guar or fiber-free EN. All patients were on mechanical ventilation, antibiotics, and catecholamine therapy. The time to reach the preconceived protein/calorie goals was 5±3 days in the prebiotic and 6±3 days in the control group. The difference was not statistically significant [25]. In another trial conducted by Tuncay et al., the prevalence of target dose achievement in 21 days intervention was 95.7% in prebiotic supplemented and 78.3% in standard EN groups. The difference was not statistically significant (*p*=0.19) [22].

#### Probiotics

Malik et al. investigated the effect of 7-day microbial cell preparation administration on the return of gut function. Time to return to normal gut function was defined as the time taken to receive a minimum of 80% of the estimated calorie for a consecutive 48-h period. They reported that patients in the treatment group achieved a faster return of gut function (3±1.75 days vs. 7±1.7 days, *p*<0.001) [26]. Ferrie et al. also investigated the effect of *Lactobacillus rhamnosus GG* on feeding intolerance in critically ill patients with established diarrhea. The frequency of patients with feeding intolerance (tolerate less than 80% of calorie goal for two consecutive days) was 11.1% in the probiotic group and 16.6% in the control group (*p*= 0.63) [27].

Based on the findings of the studies and the harvest plot, it can be concluded that majority of the studies indicated a non-significant effect for pre, pro, or synbiotics administration on target calorie achievement but the only one study that reported a significant finding had a large sample size. Therefore, it may outweigh the findings of the non-significant studies. Thus, the findings of the current studies are inconclusive.

### Effect on diarrhea

#### Prebiotics

Schultz et al. investigated the effect of pectin on the prevalence of diarrhea in a critical care setting. Diarrhea was more prevalent in the fiber-free/placebo group compared to the fiber-free/pectin group (36% vs. 9%, *p*=0.31). Diarrhea was also more prevalent in the fiber/ placebo than fiber/pectin group; however, the differences were not statistically significant (55% vs. 9%, *p*= 0.06) [19]. Majid et al. also demonstrated that fiber-enriched EN with additional prebiotic had no significant effect on the prevalence of diarrhea. The prevalence of having at least 1 day of diarrhea was 92% in the prebiotic and 90% in the placebo group (*p*=0.99). The number of days of diarrhea was 3.8±3.5 in the placebo and 3.9±4.1 in the prebiotic group (*p*= 0.94) [28]. Tuncay et al. reported that administration of prebiotic-enriched EN was associated with a significant tendency toward lower prevalence (8.7% vs. 56.5%) and faster amelioration of diarrhea (none vs. 52.2% diarrhea prevalence on day 7) [22].

In another trial, the prevalence of having at least 1 day of diarrhea was 46.1% in the prebiotic and 91.7% in the placebo group (*p*=0.03). Further, the mean frequency of days of having diarrhea was significantly lower in the fiber group. Besides, in the fiber group, diarrhea occurred in 10.8% of feeding days, compared to 31.5% in the controls (*p*<0.001) [25].

Rushdi et al. also investigated the effect of soluble guar gum on the number of liquid stools during the four days of intervention. The number of liquid stools was significantly lower at day 4 compared to day 1 in the intervention group, while it was significantly higher in the control group. The number of liquid stools on the fourth day was 1.2±0.7 in the intervention group, compared to 2.1±0.8 in the control group (*p*<0.01) [20].

#### Probiotics

In the study by Bleichner et al., 128 critically ill patients were randomized to receive *Saccharomyces Boulardii* or placebo capsules. The prevalence of diarrhea was not significantly different between the two groups. However, treatment with *S. boulardii* reduced the mean frequency of diarrhea days per feeding days from 18.9 to 14.2% (*p*= 0.006). The number of days with diarrhea was also significantly lower in the probiotic group (*p*<0.001) [29]. Barraud et al. also investigated the effect of probiotic administration on the prevalence of diarrhea. They demonstrated no significant effect of probiotic therapy on diarrhea prevalence (55.2% vs. 52.5%, *p*=0.72) [30]. In another RCT, Morrow et al. demonstrated that probiotic administration had no significant effect on the incidence of ICU-associated diarrhea. However, the number of days with ICU-associated diarrhea was significantly higher in the placebo group compared to the probiotic group (5.9±3.8 vs. 4.1±3.7, *p*=0.03) [31]. Another trial in 2010 examined the effect of probiotic VSL#3 on diarrhea among 45 critically ill patients. The mean frequency of liquid stool in the probiotic and placebo groups was 0.53±0.54 and 1.05±1.08 episodes per patient per day, respectively (*p*=0.03) [32]. Ferrie et al. also reported that critically ill patients who received probiotic had more diarrhea episodes compared to the control group, although the difference was not statistically significant. Diarrhea days in the 14-day study period was 7.22±3.63 in the probiotic and 5.72±2.88 in the synbiotic group (*p*=0.17) [27].

#### Synbiotics

Knight et al. reported the overall prevalence of diarrhea to be 5% in the synbiotic group, and 7% in the controls (*p*= 0.59) [24]. Shimizu et al. also investigated the effect of daily synbiotic therapy on infectious complications, including enteritis in the intensive care unit. Enteritis was defined as acute onset of continuous liquid stool for more than 12 h. The results showed that the incidence of enteritis was significantly lower in the synbiotic group (6.3% vs. 27.0%; *p* < 0.05) [33].

Based on the findings of the studies and the harvest plot, it can be concluded that majority of the studies indicated a significant effect for pre, pro, or synbiotics on diarrhea in terms of duration or frequency.

### Effect on length of stay

The effect of pre, pro, or synbiotics on ICU and hospital LOS was reported in 10 and 5 trials, respectively (Table 3). Malik et al. demonstrated that probiotic administration was associated with significantly lower ICU LOS [26]. Other studies found no significant difference between groups, regarding ICU or hospital LOS [19, 21, 22, 24, 27, 30–33].

**Table 3:** Reported feeding tolerance-related outcomes in RCTs evaluating the effect of pre, pro, or synbiotics on feeding tolerance of enterally fed critically ill patients

Study	Energy intake		Achieving the target calorie		Diarrhea		Length of stay	
	Intervention	Control	Intervention	Control	Intervention	Control	Intervention	Control
Bleichner et al., 1997 [17]	NR	NR	NR	NR	Prevalence: 18/64 (24%) Days w/ diarrhea per feeding days: 14.2%	Prevalence: 24/64 (38%) Days w/ diarrhea per feeding days: 18.9%	NR	NR
Schultz et al., 2000 [18]	Mean^a^	Mean^a^	NR	NR	Prevalence: 1/11 (9%)	Prevalence: 4/11 (36%)	Hospital: 34±14.7 ICU: 28±14.6	Hospital: 24.4±9 ICU: 17.2±8.2
Spapen et al., 2001 [19]	NR	NR	Time to: 5±3 days	Time to: 6±3days	Prevalence: 6/13 (46%) Days w diarrhea per feeding days: 16/148(10.8%)	Prevalence: 11/12(92%) Days w diarrhea per feeding days: 46/146 (31.5%)	NR	NR
Rushdi et al., 2004 [20]	Days 1–4	Days 1–4	NR	NR	Liquid stools day1-4	Liquid stools days 1–4	NR	NR
Knight et al., 2009 [21]	Days 1–7	Days 1–7	NR	NR	Prevalence: 7/130 (5%)	Prevalence: 9/129 (7%)	ICU: 6 (3–11)	ICU: 7 (3–14)
Frohmader et al., 2010 [22]	NR	NR	NR	NR	Frequency of liquid stools: 0.53±0.54	Frequency of liquid stools: 1.05±1.08	ICU: 7.3±5.7	ICU: 8.1±4
Barraud et al., 2010 [23]	NR	NR	NR	NR	Prevalence: 48/87(55.2)	Prevalence: 42/80(52.5)	Hospital: 26.6±22.3 ICU: 18.7±12.3	Hospital: 28.9±26.4 ICU: 20.2±20.8
Morrow et al., 2010 [24]	NR	NR	NR	NR	Prevalence: 44/70(62.9) Days w/ diarrhea: 5.9±3.8	Prevalence: 42/68(61.8) Days w/ diarrhea: 4.1±3.7	Hospital: 21.4±14.9 ICU: 14.8±11.8	Hospital: 21.7±17.4 ICU: 14.6±11.6
Ferrie and Daley, 2011 [25]	NR	NR	Prevalence 16/18 (88.8)	Prevalence 15/18(83.33)	Diarrhea duration: 7.22±3.63 Loose stool per day: 3.14±1.23	Diarrhea duration: 5.72±2.88 Loose stool per day: 3±1.2	Hospital: 54.5±31.26 ICU: 32.04±24.46	Hospital: 59.04±33.92 ICU: 29.75±18.81
Sanaie et al., 2014 [26]	Mean^a^	Mean^a^	NR	NR	NR	NR	NR	NR
Majid et al.,2014 [27]	NR	NR	NR	NR	Prevalence: 11/12 (92) Days w/ diarrhea: 3.9±4.1	Prevalence: 9/10 (90) Days w/ diarrhea: 3.8±3.5	NR	NR
Malik et al., 2016 [28]	NR	NR	Time to: 3±1.75 days	Time to: 7±1.7 days	NR	NR	ICU: 10.9±3.9	ICU: 15.8±7.8
Fazilaty et al., 2018 [29]	Mean^a^	Mean^a^	NR	NR	NR	NR	ICU: 27.55±7.8	ICU: 31.2±15.8
Shimizu et al., 2018 [30]	NR	NR	NR	NR	Incidence of enteritis: 2/35 (6.3)	Incidence of enteritis: 10/37(27)	ICU: 23 (13–43)	ICU: 28 (17–45)
Tuncay et al., 2018 [31]	Days 1 and 21	Days 1 and 21	Prevalence 22 (95.7)	Prevalence 18 (78.3)	Prevalence: 8.7%	Prevalence: 56.5%	Hospital stay <40 days: 56.6% Hospital stay ≥41 days: 43.4% ICU stay <40 days: 69.5% ICU stay ≥41 days: 43.5%	Hospital stay <40 days: 60.9% Hospital stay ≥41 days: 39.1% ICU stay <40 days: 69.5% ICU stay ≥41 days: 30.4%

*NR* Not reported; *ICU* Intensive care unit

^a^Mean energy intake was reported for the entire intervention duration

Based on the findings of the studies and the harvest plot, it can be concluded that majority of the studies indicated a non-significant effect for pre, pro, or synbiotics administration on length of stay.

## Discussion

In this systematic review, 15 randomized controlled trials were reviewed to determine the potential of pre, pro, or synbiotics administration to improve enteral feeding tolerance in tube-fed critically ill patients. Gut microbiota is a key regulator of gut function, host metabolism, and appetite. Microbial metabolites, including SCFAs, bile acids, and various neuroactive agents, interact with the GI tract and peripheral tissue through affecting the enteric nervous system and central appetite pathways or altering bile acid signaling [34]. These effects result in changes in gastric motility and emptying [35, 36], which may reduce enteral feeding intolerance. Besides, gut microbiota can influence intestinal barrier function and modulate the immune system, thus indirectly affect metabolism and eating behavior [16].

### Effect on energy intake or feed volume

We found six studies that evaluated the effect of pre, pro, or synbiotics on enteral feeding volume or energy intake. Considering the application of probiotics or synbiotics, no significant effect was reported. Only 2 of 4 studies, which used prebiotics (one soluble guar gum for 4 days and the other FOS for 21 days) in the intervention group, found significant beneficial effects [20, 22]. It should be noted that in both of these studies, patients in the intervention group received significantly more volume and energy on the first day. Therefore, it seems that the significant difference between the two groups in terms of received feed volume and energy at the end of the study may not be merely attributed to the effect of prebiotics.

### Effect on target calorie achievement

Four trials evaluated the effect of pre or probiotics on frequency or time to achieve the target calorie. All studies but one found no significant effect. In this study, probiotic administration for seven consecutive days was associated with a significantly faster return of the gut function [26]. The included studies were heterogeneous in population features, intervention, duration, eligibility criteria, and EN protocol. Thus, the conflicting results may be attributed to these factors. It is also believed that the beneficial effect of probiotics or synbiotics could be highly strain-specific.

### Effect on diarrhea

In the critical care setting, diarrhea is the most common gastrointestinal complication of EN [37], which may result in several unfavorable clinical conditions including enteral nutrition cessation and exacerbation of undernutrition [22]. Factors that contribute to the pathogenesis of diarrhea include altered physiological responses due to EN, antibiotics administration, and altered gut microbiota function [38]. Therefore, gut microbiota manipulation may be an approach for the prevention and management of diarrhea in the critical care setting. For example, gut microbiota manipulation can reverse abnormal colonic water secretion by SCFAs production [39], alter colonic motor activity [40], and interfere with pathogen colonization in the gut, which protects against diarrhea [38].

The effect of prebiotics on diarrhea was evaluated in five clinical trials [19, 20, 22, 25, 28]. Four studies investigated the effect of prebiotic on the prevalence of diarrhea. While two studies found a significant [22, 25] and one a non-significant decrease [19], the other reported a non-significant increase [28]. The number of days of diarrhea was also investigated in two studies, one of which reported a significant decrease [25], while the other found a non-significant increase [28]. The number of liquid stools was also reported to be lower in the prebiotic group in one trial [21].

It should be noticed that water-soluble fiber like pectin or guar gum exhibits antidiarrheal effect by two mechanisms: (1) production of SCFAs and maintaining gut microbiota homeostasis or (2) reuptake of water and electrolytes [41]. The beneficial effect of water-soluble fibers on SCFAs production is well documented in non-critically ill patients and healthy subjects, but it is not clearly observed in critically ill patients [41]. So, the positive effect of water-soluble fibers in the mentioned studies may be attributed to the increased reuptake of water and electrolytes, not necessarily acting as prebiotics.

Regarding the effect of probiotics on the incidence of diarrhea, two studies reported a trend towards reduced diarrhea incidence in the probiotic group [29, 31], and one reported a non-significant increase [30]. The effect of probiotic administration on diarrhea days was demonstrated in three of the included trials. Two of them reported a significant decrease in diarrhea duration [29, 31], while one reported a non-significant increase [27]. In the probiotic group, the number of liquid stools per patient per day was reported to be significantly lower in one study [32] but loose stools were non-significantly more in another study [27].

A non-significant decrease in the prevalence of diarrhea [24] and a significant decrease in the incidence of enteritis [33] were reported to be associated with synbiotic administration.

### Effect on length of stay

All but one study found no beneficial effects for gut microbiota manipulation on clinical endpoints, including LOS in hospital and ICU. A recent systematic review and meta-analysis by Manzanares et al. also showed that despite the beneficial effects of probiotic and synbiotic administration on overall infections and ventilator-associated pneumonia, these agents had no significant effect on LOS in hospital or ICU [42].

To the best of our knowledge, this systematic review was the first study to review the effect of pre, pro, and synbiotics on feeding tolerance in enterally fed critically ill patients. As we assessed relevant outcomes in a heterogeneous ICU population, our results could be attributed to a broad spectrum of critically ill patients with sepsis, trauma, or other medical conditions. Although, the inclusion of diverse patient groups in this systematic review may be considered as a limitation for interpretation of the results. There was also great diversity in the type of administered prebiotic or probiotic strains, duration of treatment, and dose. This heterogeneity also made it impossible to quantitatively evaluate the results. Furthermore, most of the included studies reported the energy intake or feeding tolerance as a secondary outcome, not mentioning the EN protocols, while the reported EN protocols were heterogeneous in other studies.

## Conclusion

Overall, the heterogeneity in studied product format, ICU patient populations, and study designs make it difficult to draw any general conclusion on the effect of pre, pro, or synbiotics on feeding tolerance of critically ill tube-fed patients. We suggest more new well-designed trials that assess feeding tolerance as a primary endpoint with a unified definition and an invariable enteral nutrition protocol that would make it possible to compare the obtained results. We have recently designed an RCT in which the main purpose is to determine the effect of synbiotics on feeding tolerance and energy homeostasis of critically ill adult patients [43]. New trials should aim to demonstrate the beneficial composition of supplements, dose, and duration to have beneficial effects. There is, moreover, a need to conduct studies that clearly establish the molecular mechanisms by which gut microbiota manipulation is attributed to feeding tolerance in critically ill patients.

## Supplementary Information

**Additional file 1:.** Preferred Reporting Items for Systematic Reviews and Meta-Analysis (PRISMA) statement

## Data Availability

The datasets generated and/or analyzed during the current study are not publicly available, but may be available from the corresponding author on reasonable request.

## Abbreviations

CCK: Cholecystokinin
EN: Enteral nutrition
FOS: Fructo-oligosaccharide
GI: Gastrointestinal
GRV: Gastric residual volume
ICU: Intensive care unit
LOS: Length of stay
PICOS: Population, intervention, comparison, outcome, and study design
PRISMA: Preferred reporting items for systematic reviews and meta-analysis
PYY: Peptide YY
RCT: Randomized controlled trials
SCFAs: Short-chain fatty acids
